# Cytoplasmic incompatibility in the semivoltine longicorn beetle *Acalolepta fraudatrix* (Coleoptera: Cerambycidae) double infected with *Wolbachia*

**DOI:** 10.1371/journal.pone.0261928

**Published:** 2022-01-14

**Authors:** Takuya Aikawa, Noritoshi Maehara, Yu Ichihara, Hayato Masuya, Katsunori Nakamura, Hisashi Anbutsu

**Affiliations:** 1 Tohoku Research Center, Forestry and Forest Products Research Institute, Morioka, Iwate, Japan; 2 Department of Forest Entomology, Forestry and Forest Products Research Institute, Tsukuba, Ibaraki, Japan; 3 Kansai Research Center, Forestry and Forest Products Research Institute, Kyoto, Japan; 4 Department of Mushroom Science and Forest Microbiology, Forestry and Forest Products Research Institute, Tsukuba, Ibaraki, Japan; 5 Computational Bio Big-Data Open Innovation Laboratory, National Institute of Advanced Industrial Science and Technology, Shinjuku-ku, Tokyo, Japan; 6 Bioproduction Research Institute, National Institute of Advanced Industrial Science and Technology, Tsukuba, Ibaraki, Japan; 7 Research Organization for Nano and Life Innovation, Waseda University, Shinjuku-ku, Tokyo, Japan; USDA Agricultural Research Service, UNITED STATES

## Abstract

*Wolbachia* are obligatory endosymbiotic α-proteobacteria found in many arthropods. They are maternally inherited, and can induce reproductive alterations in the hosts. Despite considerable recent progress in studies on the associations between *Wolbachia* and various taxonomic groups of insects, none of the researches have revealed the effects of *Wolbachia* on longicorn beetles as the host insect. *Acalolepta fraudatrix* is a forest longicorn beetle that is distributed in East Asia. In this study, the relationship between *Wolbachia* and *A*. *fraudatrix* was investigated. Out of two populations of *A*. *fraudatrix* screened for *Wolbachia* using the genes *ftsZ*, *wsp*, and 16S rRNA, only one of the populations showed detection of all three genes indicating the presence of *Wolbachia*. Electron microscopy and fluorescent *in situ* hybridization also confirmed that the *A*. *fraudatrix* population was infected with *Wolbachia*. Sequencing the *wsp* genes derived from single insects revealed that two strains of *Wolbachia* coexisted in the insects based on the detection of two different sequences of the *wsp* gene. We designated these strains as *w*Fra1 and *w*Fra2. The bacterial titers of *w*Fra1 were nearly 2-fold and 3-fold higher than *w*Fra2 in the testes and ovaries, respectively. The two strains of *Wolbachia* in the insects were completely eliminated by rearing the insects on artificial diets containing 1% concentration of tetracycline for 1 generation. Reciprocal crosses between *Wolbachia*-infected and *Wolbachia*-uninfected *A*. *fraudatrix* demonstrated that only eggs produced by the crosses between *Wolbachia*-infected males and *Wolbachia*-uninfected females did not hatch, indicating that *Wolbachia* infecting *A*. *fraudatrix* causes cytoplasmic incompatibility in the host insect. This is the first report showing the effect of *Wolbachia* on reproductive function in a longicorn beetle, *A*. *fraudatrix*.

## Introduction

The members of the genus *Wolbachia* are common intracellular symbionts that are maternally inherited in arthropods and filarial nematodes, and they are estimated to infect up to 60% of insects [1–6]. They are capable of manipulating host reproduction by causing cytoplasmic incompatibility (CI), parthenogenesis, male killing or feminization [7–11]. Of these reproductive alterations caused by *Wolbachia*, CI is the most common, and has been observed in many arthropod species [12]. The effect of CI on reproduction is typically unidirectional. Crosses between infected males and uninfected females are incompatible yielding fertilized but unviable eggs, whereas other crosses are compatible and produce normal progeny. CI leads to a reproductive advantage of infected over uninfected females, and infection frequencies therefore rise over generations [13]. The ability of *Wolbachia* to cause these reproductive alterations drives its efficient and rapid spread in host populations [5, 14, 15].

There are many reviews summarizing the state of knowledge about *Wolbachia* infection groups of insects, including Heteropteran bugs [16], springtails [17], ants [18] wasps [19], butterflies [20], and mosquitoes [21]. Despite this progress in studies on *Wolbachia* infection in insects, knowledge about its interaction with species in the order Coleoptera is still sparse [22]. Among the Coleopteran insects, *Wolbachia* has been detected in 81 species of chrysomelid and 49 species of curculionid beetles, and research is relatively advanced in these two families [22]. However, there have been few reports on *Wolbachia* detection in cerambycid beetles. To our knowledge, only two cases have been reported so far. The first is *Monochamus sartor*, which is distributed throughout Europe, and in Kazakhstan, Mongolia, North Korea, South Korea and Hokkaido in Japan [23]. Four strains of *Wolbachia* were found from three European populations of *M*. *sartor*, but the impact of these strains of *Wolbachia* on the host insect is unknown [24]. The second species investigated is *Monochamus alternatus*, which inhabits Japan, the Korean Peninsula, China, and Taiwan [23]. This insect is the longicorn beetle, notorious as a vector of the pinewood nematode, *Bursaphelenchus xylophilus*, which causes pine wilt disease [25–27]. The relationship between *Wolbachia* and this insect is unique. The beetle is not infected with *Wolbachia* as a bacterium, but carries many *Wolbachia* genes on an autosome, indicating that lateral gene transfer has occurred from *Wolbachia* to *M*. *alternatus* [28, 29]. Very few reports of the detection of *Wolbachia* in the cerambycid insects have been published, compared to those related to other insect taxa, and there has been no research into the effect of *Wolbachia* on the insects.

*Acalolepta fraudatrix* is a forest longicorn beetle that inhabits Japan, the Korean Peninsula and China [23]. This beetle has extremely wide host adaptability, and can utilize not only coniferous trees such as pine and fir, but also broad-leaved trees such as oak and cherry, as hosts [30]. It is distributed all over Japan, and is recognized as a common species that can be found in any forest. However, rearing *A*. *fraudatrix* for successive generations is very difficult, because of its long generation time [31] and the lack of established methods for rearing in the laboratory. Although this insect is challenging to work with as experimental material, such research is also rewarding.

In this research, we studied two populations of *A*. *fraudatrix* from Japan. Diagnostic polymerase chain reaction (PCR) detection of *Wolbachia* genes was performed on the two populations, and *Wolbachia* genes were detected in one of the populations. The major aims in this study were to clarify 1) whether the population of *A*. *fraudatrix* is infected with one or more strains of *Wolbachia* and 2) whether *Wolbachia* infecting *A*. *fraudatrix* causes reproductive manipulation in the host insect. A decade of detailed research has revealed that the population of *A*. *fraudatrix* carries two strains of *Wolbachia*, which cause CI in the host insect. This paper is the first to report that *Wolbachia* affects the reproductive function of an insect belonging to the family cerambycidae.

## Materials and methods

### Insect material

The collection of insect material in the field was carried out with the permission of the Aomori Prefectural Government and the Iwate Prefectural Government. One hundred and ninety larvae of final instar of *A*. *fraudatrix* were collected from a *Pinus thunbergii* forest in the town of Fukaura, Aomori Prefecture (40°26’10.7"N 139°56’30.8"E), and 90 larvae were collected from a *Pinus densiflora* forest in the town of Shiwa, Iwate Prefecture (39°36’04.2"N 141°12’15.3"E) in north-eastern Japan in April, from 2010 to 2020 (S1 Fig). *A*. *fraudatrix* populations that inhabit in these areas show a semivoltine life cycle [31]. Pine wilt disease has spread over the last decade around these survey sites, and trees infected with the nematodes, as well as healthy trees, were felled and cut into logs to control the disease. The *A*. *fraudatrix* larvae were obtained from the pine logs. They were individually placed in 15 ml test tubes and transported to Tohoku Research Center, Forestry and Forest Products Research Institute, Morioka City, Iwate Prefecture, Japan, and stored at 5–10°C. Each larva was placed on moistened filter paper in a petri dish (6 cm in diameter) and incubated at 25°C for one month before the start of experiments, to allow the larvae to develop into adults. The adult insects after pupation and eclosion were used in the following experiments.

### Artificial diets

Two artificial diets established for rearing *M*. *alternatus* [28, 32] were used for rearing *A*. *fraudatrix* larvae and adults. In diet Ⅰ, a mixture of 4.0 g of milled inner bark of *P*. *densiflora*, 6.6 g of artificial silkworm diet (Silkmate 2M, Norsan Co., Kanagawa, Japan), and 0.8 g of dried yeast (EBIOS, Asahi Group Foods, Ltd., Tokyo, Japan) was kneaded with 8.6 ml of distilled water, an approach modified from that described in [28]. Diet Ⅱ was composed of 1.6 g of current and one-year-old needles of *P*. *densiflora* dried at 70°C for one day and milled into powder, 5.4 g of Silkmate 2M, 0.6 g of EBIOS, and 12.4 ml of distilled water [32]. Approximately 20 g of each diet was placed into a 50 ml Erlenmeyer flask. Each flask was plugged with a silicone-rubber stopper (Silicosen, Shin-Etsu Polymer Co., Ltd., Tokyo, Japan) and autoclaved at 121°C for 20 min.

Artificial diet containing an antibiotic (tetracycline) was modified from artificial diet I as follows. A mixture of 6.6 g of Silkmate 2M, 0.8 g of EBIOS and 4.0 g milled inner bark of *P*. *densiflora* was kneaded with 7.6 ml of distilled water, and 19.0 g of food clod was prepared in a 50 ml flask. The flask was autoclaved at 121°C for 20 min, and after cooling down, 1 ml of 10% or 20% tetracycline solution was added to each flask. Hence, artificial diet I containing 0.5% or 1.0% (wt/wt) tetracycline was prepared.

### DNA extraction

Ovary and testis were chosen as reproductive tissues, and thoracic muscle was chosen as the representative of somatic tissues. Ovaries, testes and thoracic muscles were dissected from adult insects of the Fukaura and Shiwa populations, and fat bodies were prepared from final instar larvae of the Fukaura population. These tissues were individually subjected to DNA extraction using a DNeasy Blood and Tissue Kits (Qiagen, Hilden, Germany). The concentration of the extracted DNA was measured using Qubit^®^ 2.0 Fluorometer and dsDNA HS Assay kit (Invitrogen, Waltham, USA). The purified DNA liquid was used as a template for PCR. To confirm successful DNA extraction, we amplified a 0.7 kb fragment of mtDNA that included portions of the genes for cytochrome *c* oxidase II and tRNA^Leu-UUR^ of longicorn beetles, as a control [33].

### Diagnostic PCR detection and sequencing

Diagnostic PCR was used to search three *Wolbachia* genes using *Wolbachia* universal primer sets from the insect DNA samples: a 0.75 kb fragment of the *ftsZ* gene with the primers ftsF (5′-GTA TGC CGA TTG CAG AGC TTG-3′) and ftsR (5′-GCC ATG AGT ATT CAC TTG GCT-3′) [34]; a 0.6 kb fragment of the *wsp* gene with the primers 81F (5′-TGG TCC AAT AAG TGA TGA AGA AAC-3′) and 691R (5′-AAA AAT TAA ACG CTA CTC CA-3′) [35]; a 0.89 kb fragment of the 16S rRNA gene with the primers 99F (5′-TTG TAG CCT GCT ATG GTA TAA CT-3′) and 994R (5′-GAA TAG GTA TGA TTT TCA TGT-3′) [9]. The reaction was carried out in 20 μl of reaction mixture containing 2 μl of extracted DNA solution, 2.5 μl of 4 μM each primer, 10 μ1 of GoTaq^®^ Hot Start Green Master Mix (Promega, Madison, USA), and 3 μl of ultrapure water in a 0.2 μl microtube. The PCR conditions were as follows: 95°C for 2 min followed by 35 cycles at 94°C for 1 min, 52°C for 1 min and 72°C for 1 min, and a final elongation step at 72°C for 2 min. DNA samples extracted from adults of *Callosobruchus chinensis* infected with *Wolbachia* [36] and ultrapure water were used as positive and negative controls, respectively. PCR diagnoses for other endosymbiotic bacteria, *Rickettsia*, *Spiroplasma*, *Arsenophonus* and *Cardinium*, which have been known to cause reproductive manipulation in the host insects [37–40] were also performed using the primers and conditions listed in S1 Table [41]. As positive controls, DNA samples extracted from each individual of *Mallada desjardinsi* infected with *Rickettsia* and *Spiroplasma* [42], *Sogatella furcifera* infected with *Cardinium* [43], and *Trialeurodes vaporariorum* infected with *Arsenophonus* were used.

The PCR products were electrophoresed on agarose gels, stained with ethidium bromide, and observed on an ultraviolet trans-illuminator. Sequencing of the PCR products was conducted using BigDye Terminator v. 3.1 Cycle Sequencing Kits using the ABI 3100 DNA sequencer (Applied Biosystems, Waltham, USA).

### Specific PCR detection of two types of *wsp* gene of *Wolbachia*

A 0.5 kb fragment of each of the two *wsp* genes of *Wolbachia* were detected using the specific forward PCR primers wspwFra1_F (5′-GTT GAT GGT ATT ACC TAT AAG AA-3′) and wspwFra2_F (5′-GTT GAT GGT ATT GCA CAT AAA TC-3′) in combination with the universal reverse primer 691R for the *wsp* gene (S2 Fig). The PCR temperature profile was the same as that used for the *Wolbachia* diagnosis.

### Molecular phylogenetic analysis

DNA extracted from two males and two females of *Wolbachia*-infected (*W*^+^) population was used as a template for *Wolbachia* multilocus sequence typing (MLST). MLST genes (*gatB*, *coxA*, *hcpA*, *ftsZ*, and *fbpA*) were amplified using specific primer sets described in S1 Table [44]. The composition of the PCR reaction mixture was the same as above. We carried out PCR following conditions: 95°C for 2 min followed by 35 cycles at 94°C for 30 sec, the optimal annealing temperature (see S1 Table) for 30 sec and 72°C for 1 min, and a final elongation step at 72°C for 2 min. The concatenated sequences of the five MLST genes were used in molecular phylogenetic analysis. Clustal W [45] was used to generate multiple alignments of the sequences from *A*. *fraudatrix* and those obtained by Baldo et al. [44]. By using the program MEGA7 [46], GTR + G + I was selected as the best fit model and a maximum-likelihood tree was then constructed. Bootstrap values were obtained by generating 1,000 bootstrap replications.

### Fluorescent *in situ* hybridization (FISH)

*W*^+^ adult females were reared for two months on artificial diet I, then their ovaries were dissected in phosphate buffered saline (PBS) (1.9 mM NaH_2_PO_4_, 8.1 mM Na_2_HPO_4_, 175 mM NaCl, pH 7.4) and soaked in a 4% paraformaldehyde / PBS solution for 4 h. They were washed with PBS solution and stored in 70% ethanol for one week. Then they were treated with ethanol, xylene, and paraffin using the usual method, and finally embedded in paraffin. The paraffin-fixed tissues were sliced at a thickness of 5 μm using a microtome, and then deparaffinized with ethanol and xylene on glass slides. The *Wolbachia* symbiont was detected with two oligonucleotide probes Wol3 (5′-TCC TCT ATC CTC TTT CAA TC-3′) and Wol4 (5′-GAG TTA GCC AGG ACT TCT TC-3′) targeted to *Wolbachia* 16S rRNA, which were labeled with Alexa647 [47]. Fifty nanograms of DNA of each probe was dissolved in 10 μl of hybridization solution (20% formamide, 0.9 M NaCl, 20 mM Tris-HCl pH7.2, 0.01% SDS) and applied to the samples. After hybridization at 46°C for 2 h, and stringent washing, the samples were stained using DAPI, and the hybridization signals were visualized using a CW4000 fluorescent microscope (Leica, Wetzlar, Germany) and image analysis software.

### Electron microscopy

The experiment was conducted using a method modified from Gill et al. [48]. Dissected ovaries and testes from *W*^+^ adult insects just after eclosion were prefixed and stored in 2% glutaraldehyde in phosphate buffer (0.1M, pH7.3) at 4°C, and then postfixed in 2% osmium tetroxide overnight. The tissues were rinsed three times in the buffer, dehydrated in an ethanol series, and embedded in LR-white Medium resin (Sigma–Aldrich, St. Louis, USA). Ultrathin sections were cut with an MT-2B Sorvall ultramicrotome (Sigma–Aldrich), mounted on Formvar-coated copper grids (Nisshin EM Co. Ltd., Tokyo, Japan), stained with uranyl acetate and lead citrate, and observed using a transmission electron microscope (JEM-2000EX; Jeol, Tokyo, Japan) at 200 kV.

### Quantitative PCR

Real-time fluorescence detection quantitative PCR was performed using the StepOnePlus Real-time PCR System (Applied Biosystems). Dissected ovaries, testes and thoracic muscles from *W*^+^ insects were washed with PBS solution and then subjected to DNA extraction. To quantify the two types of sequences of the *wsp* gene (each 0.16 kb fragment) separately, highly specific amplifying primers were designed manually based on the differences between the two sequences: wspwFra1_F and QwFra1_R (5′-TTA AAT GCT GCA CCT GTA ACA-3′); wspwFra2_F and QwFra2_R (5′-TGT ATC ACC TAC TAC ATC TGC A-3′) (S2 Fig). The reaction was performed in a total volume of 20 μl containing 2 μl DNA template, 2.5 μl of each primer (10 μM), 10 μl of PowerUp^TM^ SYBR^®^ Green Master Mix (Applied Biosystems) and 3 μl of ultrapure water. Ultrapure water was used as a negative control. Copy numbers of the two *wsp* genes in the samples were quantified using the primers under a temperature profile of 45 cycles of 95°C for 15 sec, 52°C for 30 sec and 72°C for 1 min. To check the specificity of the amplification, melt curves were generated at the end of each run, as follows: 95°C for 15 sec, 60°C for 1 min, and 95°C for 15 sec. The sequences of the two *wsp* genes were synthesized and subcloned into pEX-K4J1 plasmid by Eurofins Genomics K. K. (Tokyo, Japan). Standard curves were drawn using the plasmid samples that contained the *wsp* genes at concentrations of 10^8^, 10^7^, 10^6^, 10^5^, 10^4^, 10^3^ and 10^2^ copies per 2 μl. The number of copies of the *wsp* gene per nanogram DNA was estimated by dividing the number of gene copies in each sample subjected to quantitative PCR by the total DNA concentration of the sample. Quantification of each sample was repeated in triplicate and the average value was used in statistical analysis.

### Antibiotic treatment

Removal of *Wolbachia* from *A*. *fraudatrix* by tetracycline was attempted to obtain *Wolbachia*-free adults for use in CI examination. *W*^+^ adult insects just after eclosion were individually reared on artificial diet II in the flasks for one month, to allow sexual maturation. Then, a male and a female were placed in a plastic container (20 × 10 × 6 cm) with dried and fresh leaves of *Morus bombycis* as food for adult insects. After mating, females were allowed to oviposit for nine days on bolts of *Larix kaempferi* (5 cm in diameter and 43 cm long), which had been felled about one month prior, in plastic containers (38 × 25 × 28 cm). The oviposited bolts were put into plastic bags, stored at 25°C for two weeks, and then peeled with a knife and forceps to obtain the first or second instar larvae. The collected larvae were weighed, washed with sterilized distilled water, and then 20, 20 and 10 larvae were individually placed on artificial diet I containing 0.5%, 1.0%, or no tetracycline, respectively. After rearing for three months at 25°C, the final instar larvae in the flasks were kept at 5°C for three months, then were maintained at 25°C for pupation and adult eclosion. Four or five days after eclosion, the adult insects were dissected, and the ovaries and testes were removed from females and males, respectively. These tissues were subjected to DNA extraction and detection of the two types of *Wolbachia* using PCR.

### Examination of CI

To detect CI caused by *Wolbachia* in *A*. *fraudatrix*, mating experiments were performed with *W*^+^ and *Wolbachia*-uninfected (*W*^−^) adult insects. The adults after eclosion were individually reared on artificial diet II in 50 ml flasks for one to two months. After sexual maturation, four patterns of crosses were attempted. Cross 1 was *W*^+^ males × *W*^+^ females, Cross 2 was *W*^−^ males × *W*^−^ females, Cross 3 was *W*^−^ males × *W*^+^ females, Cross 4 was *W*^+^ males × *W*^−^ females. The number of the pairs in four reciprocal crosses was as follows: Cross 1, 13 pairs; Cross 2, 16 pairs; Cross 3, 12 pairs; Cross 4, 14 pairs. A male and a female were coupled in a plastic container, and then oviposited bolts of *L*. *kaempferi* were obtained, as described above. The bolts were put into individual plastic bags, and kept at 25°C for four weeks. Then the bolts were peeled with a knife and forceps, and the numbers of larvae and eggs that had not hatched were counted.

### Statistical analysis

Statistical analysis was performed using BellCurve for Excel ver. 2.14 (SSRI Co., Ltd., Tokyo, Japan). For the statistical analysis, normality and homoscedasticity tests were performed to decide whether to use parametric or non-parametric tests. The proportions of hatched eggs to total eggs oviposited was compared among four cross tests using *W*^+^ and *W*^−^ insects using one-way ANOVA and Tukey–Kramer’s multiple comparison test. The percentage of hatched eggs was transformed to an angle by arcsine transformation before analysis [49]. Kruskal–Wallis one-way analysis was performed to compare the weights of larvae, before they were supplied with artificial diets with different antibiotic concentrations. The differences in the bacterial titers between the two strains of *Wolbachia* in each tissue type of the adult insects were analyzed using Mann–Whitney *U*-test.

## Results

### Detection of *Wolbachia* genes from *A*. *fraudatrix* populations

Twenty-three adults (nine males and 14 females) and 13 larvae of *A*. *fraudatrix* from the Fukaura population, and 10 adults (six males and four females) from the Shiwa population were subjected to diagnostic PCR detection of *Wolbachia* using the *fts*Z, *wsp*, and 16S rRNA genes, and four other symbiotic bacteria, *Rickettsia*, *Spiroplasma*, *Arsenophonus* and *Cardinium* using the 16S rRNA gene. The three *Wolbachia* genes were detected in the testes and ovaries of all adults and fat bodies of all larvae of the Fukaura population (*W*^+^), whereas no gene was detected in individuals of the Shiwa population (*W*^−^) (S3 Fig, S2 Table). The genes of the four symbiotic bacteria other than *Wolbachia* were not detected in any of the individuals from either population (S2 Table).

### Identification of two types of *Wolbachia* genes

Direct sequencing of the PCR products of the *fts*Z, *wsp*, and 16S rRNA genes derived from single insects showed overlapping sequence spectra, suggesting that multiple strains of *Wolbachia* coexist in *A*. *fraudatrix*. To further characterize *Wolbachia* in *A*. *fraudatrix*, we cloned the *ftsZ*, *wsp* and 16S rRNA gene fragments amplified from a single *W*^+^ male. Two types of sequences were identified in each of the three genes. Although the difference in the sequences between the two types was only one base substitution in the *ftsZ* and 16S rRNA genes (GenBank accession numbers *fts*Z (728 bp): LC599919, 16S (852 bp): LC599920), it was larger in the *wsp* gene. Hence, we decided to distinguish between the two types of *Wolbachia* by using the sequence of the *wsp* gene. Hereafter, we call the two types of *Wolbachia w*Fra1 and *w*Fra2 (*wsp* of *w*Fra1 (558 bp): LC552069, *wsp* of *w*Fra2 (546 bp): LC552070). The DNA samples of all 23 adults and 13 larvae from the Fukaura population were subjected to specific PCR to detect the strains *w*Fra1 and *w*Fra2 separately. Both strains were detected in all individuals of the Fukaura population (S2 Table). Direct sequencing of the PCR products of two males and two females yielded unambiguous sequences, indicating that the two *wsp* sequences of the strains *w*Fra1 and *w*Fra2 were amplified separately.

### Molecular phylogenetic relationship of two strains of *Wolbachia*

**Fig 1:**
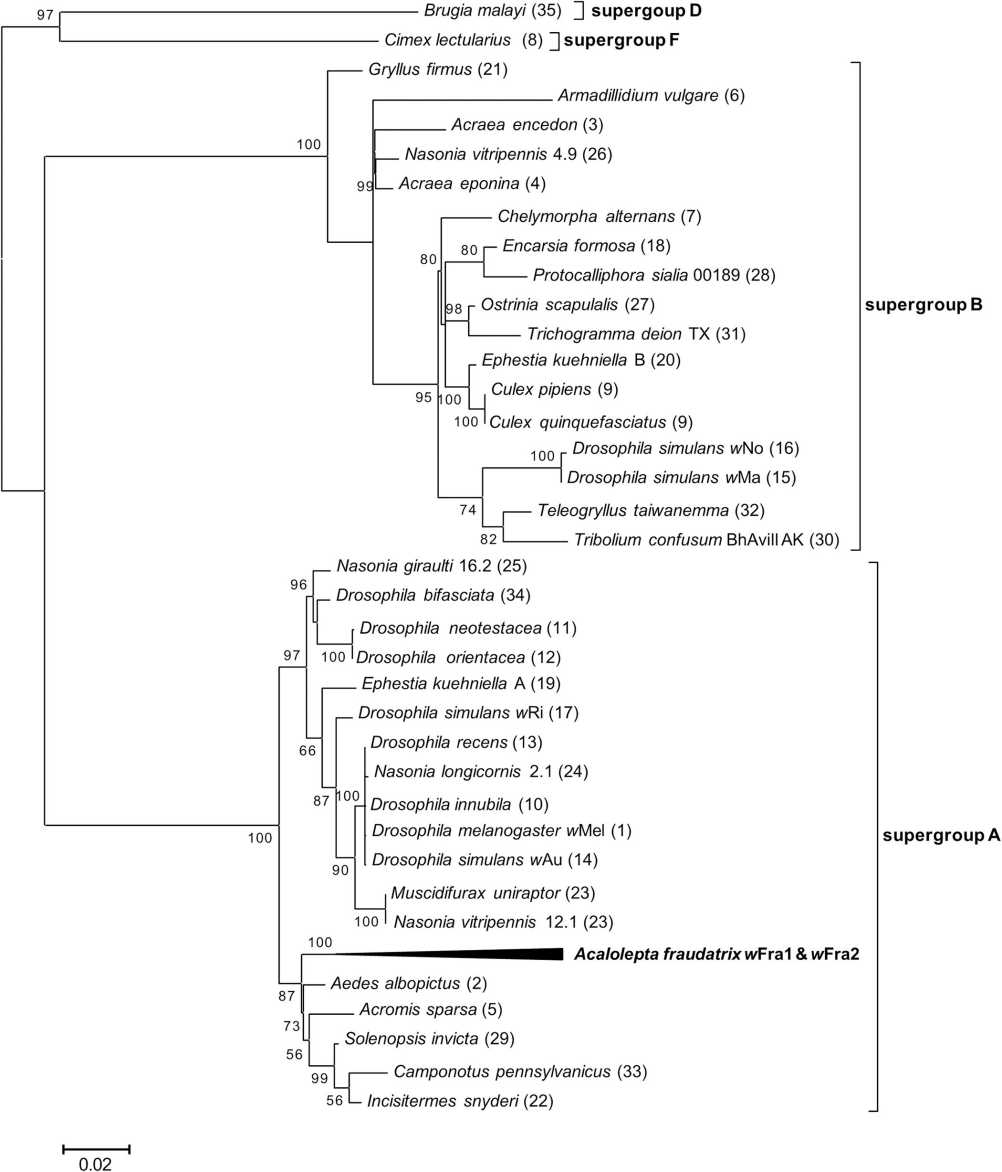
Phylogeny of *Wolbachia* based on the concatenated data set for the five MLST gene sequences. Phylogenetic analysis was conducted using two strains (*w*Fra1 and *w*Fra2) from this study, and 37 strains from Baldo et al. [44] using maximum likelihood method based on the GTR + G + I model. Since it was not possible to distinguish between the strains *w*Fra1 and *w*Fra2 in the *ftsZ* gene, the subtrees of both strains were compressed. Bootstrap values exceeding 50% are shown (1,000 replicates). A total of 2,013 aligned nucleotide sites were subjected to the analysis. The numbers in parentheses indicate the sequence type in *Wolbachia* MLST database.

Direct sequencing of the PCR product of each of the *gatB*, *coxA*, *hcpA* and *fbpA* genes showed a single sequence, but there was one base substitution in the *ftsZ* gene, as mentioned above (GenBank accession numbers *gatB* (429 bp): LC653332, *coxA* (446 bp): LC653333, *hcpA* (476 bp): LC653334, *fbpA* (467 bp): LC653336, *ftsZ* (481 bp): LC653335). There were no differences in the sequences of these genes among individuals. The MLST gene sequences of *w*Fra1 and *w*Fra2 were subjected to molecular phylogenetic analysis. The evolutionary lineages of *Wolbachia* were designated a “supergroup” [35, 50–52], according to the definitions, the two strains *w*Fra1 and *w*Fra2 of *Wolbachia* derived from *A*. *fraudatrix* were placed in supergroup A (Fig 1).

### Histological observations of *Wolbachia* using FISH and electron microscopy

**Fig 2:**
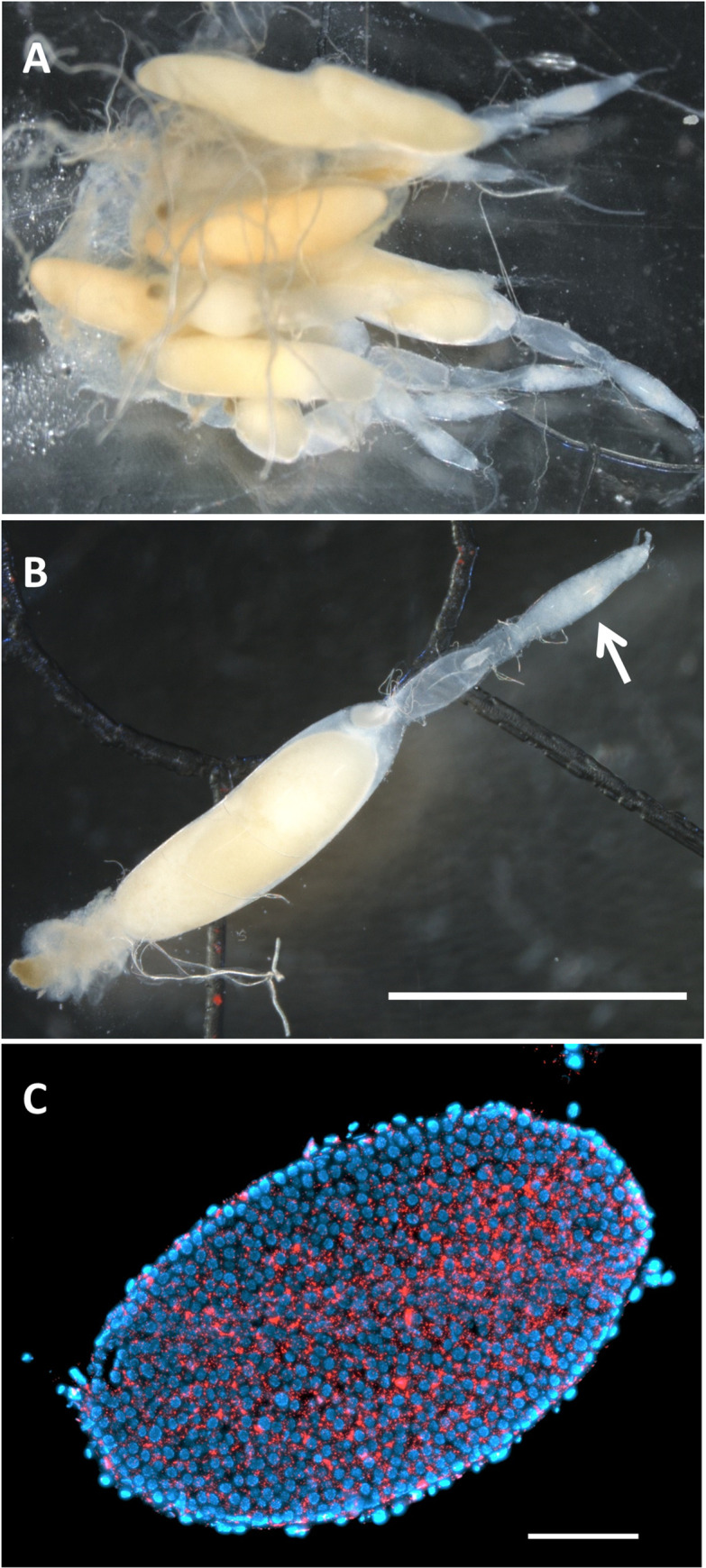
Fluorescent microscopy images supporting the presence of *Wolbachia* in ovarioles of *A*. *fraudatrix*. A: an ovary of a sexually mature female. B: an ovariole (arrow indicates germarium). C: *Wolbachia* distribution in a cross section of a germarium. *Wolbachia* are visualized in red and insect nuclei are blue (C). Scale bars = 5 mm for B, 50 μm for C.

To visualize *Wolbachia* in tissues of *A*. *fraudatrix*, histological analyses were performed using FISH and electron microscopy. In FISH analysis, the two strains of *Wolbachia* were observed collectively, not differentially, to obtain a strong *Wolbachia* signal. An ovary consisting of several ovarioles was retrieved from a sexually mature female (Fig 2A). The ovarioles were individually observed under a light microscope (Fig 2B). The germariums of the ovarioles were subjected to fluorescent imaging analysis. *Wolbachia* signals were detected in all germaria observed, and seemed to be densely present, filling the gaps in the tissues (Fig 2C).

**Fig 3:**
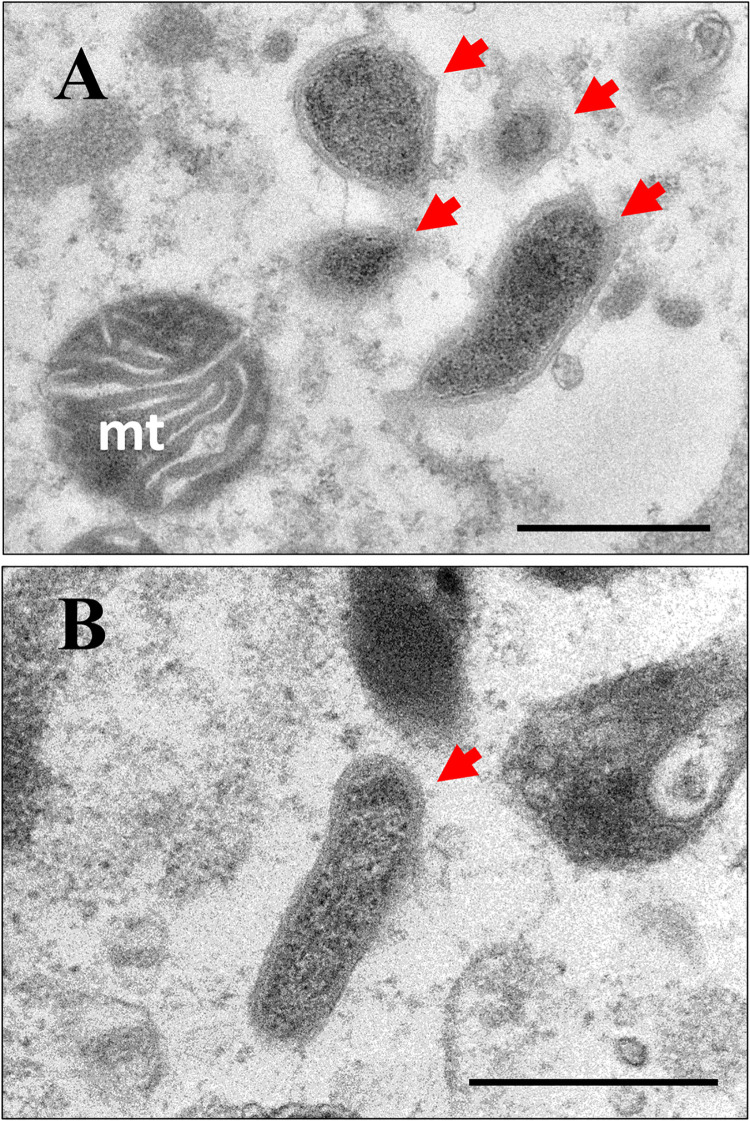
Electron micrographs of *Wolbachia* in the ovariole and testis of *A*. *fraudatrix*. A: ovariole, B: testis. The arrows indicate *Wolbachia*-like cells. Mt: mitochondrion. Scale bar = 0.5 μm.

Fig 3 shows electron microscopic images of *Wolbachia* in the ovariole and testis of *W*^+^ adults. Many objects with a double membrane structure, characteristic of bacteria, were observed intracellularly.

### Quantification of two strains of *Wolbachia*

**Fig 4:**
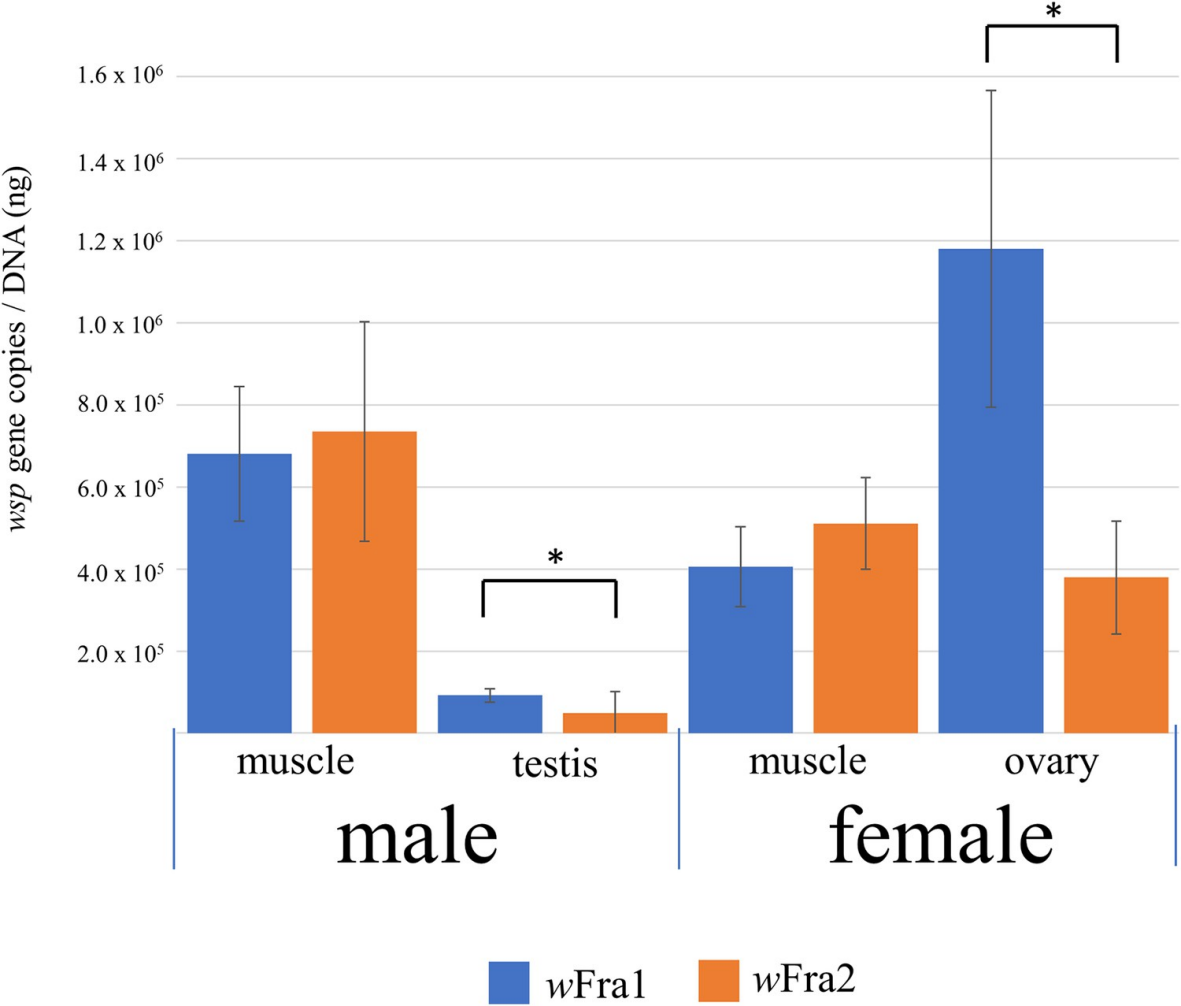
Quantification of two strains of *Wolbachia* in different tissues of *A*. *fraudatrix*. Titers of the *wsp* genes were evaluated by quantitative PCR in terms of *wsp* gene copies per nanogram of total DNA in each sample. The means and standard deviations of 10 measurements are shown for the male thoracic muscle, testis, female thoracic muscle, and ovary. Asterisks indicate statistically significant differences at the 1% level (Mann–Whitney *U*-test).

To quantitatively investigate the localization of *w*Fra1 and *w*Fra2 in tissues of adult *A*. *fraudatrix*, the ovaries, testes and thoracic muscles of 10 females and 10 males collected in the field were subjected to quantitative PCR analysis. Direct sequencing of the PCR products amplified with the *w*Fra1 specific primers (wspwFra1_F—QwFra1_R) and the *w*Fra2 specific primers (wspwFra2_F—QwFra2_R) showed clear and noise-free traces of the sequences, indicating that the *wsp* sequences of *w*Fra1 and *w*Fra2 were successfully amplified separately by these primer sets. There was no statistical difference in the titers between the strains *Wolbachia w*Fra1 and *w*Fra2 in muscle, irrespective of sex (Mann–Whitney *U*-test, male: *P* = 1.000, female: *P* = 0.052). However, the testes and ovaries contained *w*Fra1 titers nearly twice and three times as great as those of *w*Fra2, respectively, and the differences were statistically significant (Mann–Whitney *U*-test, testis: *P* = 0.002, ovary: *P* < 0.001) (Fig 4).

### Elimination of *Wolbachia* by antibiotic treatment

To determine the concentration of antibiotic needed to eliminate *Wolbachia* from the host insects, 20, 20 and 10 larvae were reared on artificial diets containing 0.5%, 1.0% and 0% (control) tetracycline, respectively until they developed into adults. Before being placed on the artificial diets, there were no statistically significant difference in the weights of the insect larvae among the three treatments (average ± SD = 24.7 ± 15.2 mg in 0.5% treatment, 22.6 ± 13.9 mg in 1.0% treatment, 24.5 ± 10.1 mg in control) (Kruskal–Wallis test, *P* = 0.654), indicating that the developmental stage of the larvae used in this experiment did not differ among the treatments. Of the larvae used in this experiment, 11/20 (three male and eight female), 7/20 (three male and four female) and 6/10 (one male and five female) individuals developed into adults in the 0.5%, 1.0%, and control treatments, respectively. Strains *w*Fra1 and *w*Fra2 of *Wolbachia* disappeared completely from the bodies of the insects following 1.0% treatment (Table 1) (S4 Fig). However, it was difficult to efficiently produce many *Wolbachia*-free adults with the artificial diets containing 1% concentration of tetracycline (Table 1).

**Table 1:** Proportion of *A*. *fraudatrix* adults whose *Wolbachia* was detected by diagnostic PCR after antibiotic treatment.

	*Wolbachia* strain	
Tetracycline concentration	*w*Fra1	*w*Fra2
0.5% (11)	9% (1)	0%
1.0% (7)	0%	0%
0.0% (6)	100% (6)	100% (6)

Values in parenthesis represent the numbers of insects.

### CI in *A*. *fraudatrix* caused by *Wolbachia*

**Fig 5:**
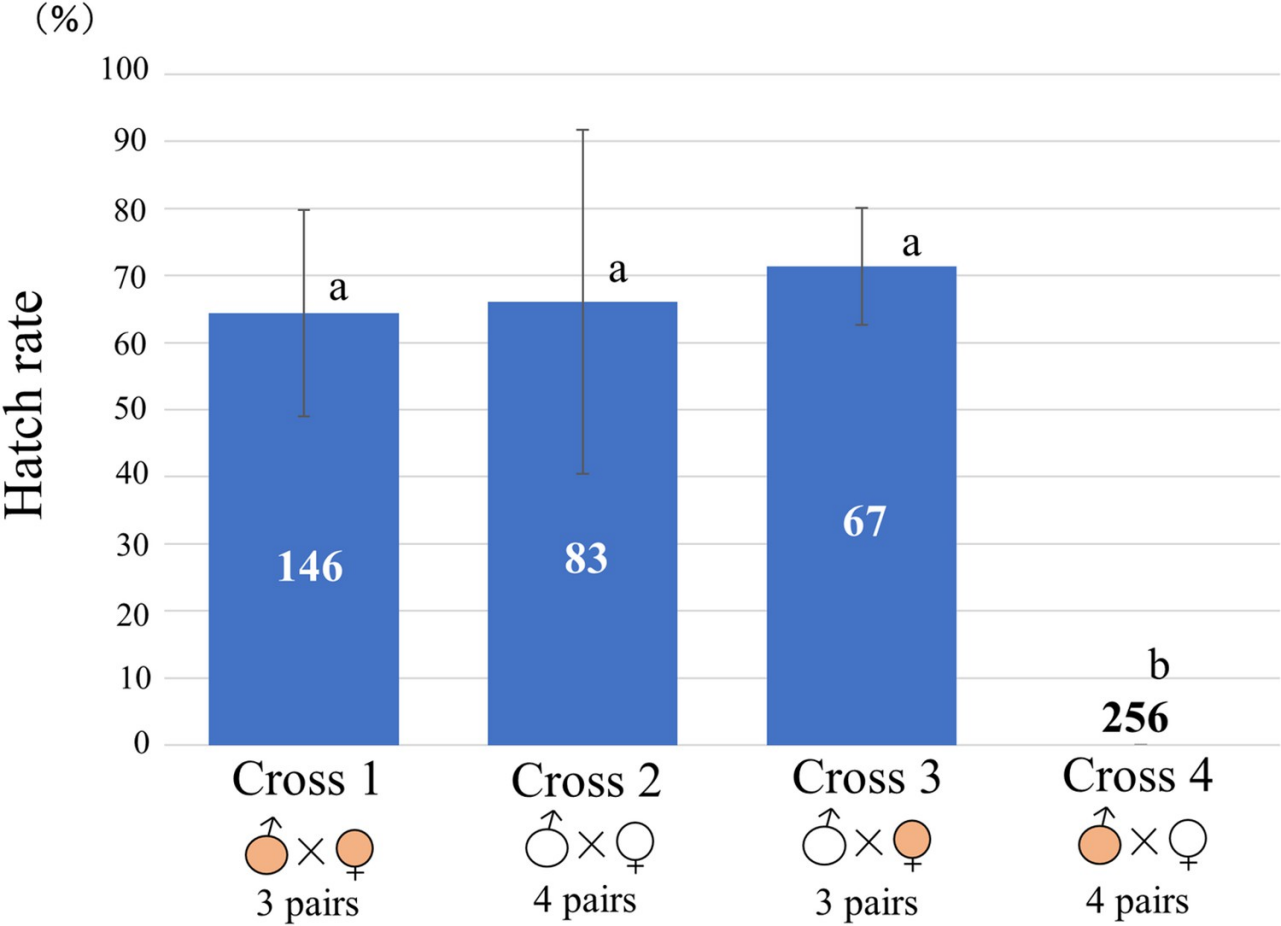
Hatch rates of eggs produced by reciprocal crosses using *W*^+^ and *W*^−^ *A*. *fraudatrix*. Means and standard deviations are shown for four crosses. The number in each column indicates the total number of eggs produced by the pairs assigned to each cross. Colored symbols of male and female indicate infection with *Wolbachia*. a and b indicate statistically significant differences at the 1% level (Tukey–Kramer’s multiple comparison test).

Since it was difficult to obtain many *Wolbachia*-free adults using antibiotic treatment, the adults from the Fukaura and Shiwa populations collected in the field were used in CI examination as *W*^+^ and *W*^−^, respectively. A total of 55 mating pairs were prepared (Cross 1: 13 pairs ♂*W*^+^ × ♀*W*^+^, Cross 2: 16 pairs ♂*W*^−^ × ♀*W*^−^, Cross 3: 12 pairs ♂*W*^−^ × ♀*W*^+^, Cross 4: 14 pairs ♂*W*^+^ × ♀*W*^−^). Of these pairs, three from Cross 1, four from Cross 2, three from Cross 3, and four from Cross 4, that oviposited more than 10 eggs were used for analysis. We registered 100% embryonic lethality in Cross 4 (0 hatched from 256), whereas in the other crosses the hatch rates were approximately 60–70% (Fig 5). Therefore, the difference in the hatch rate between the incompatible Cross 4 versus other control crosses was significant (Tukey–Kramer’s multiple comparison test, *P* < 0.001) that indicated strong CI.

## Discussion

On the basis of the results of the molecular biological and histological analyses performed in this study, we inferred that the Fukaura population of *A*. *fraudatrix* was superinfected with strains *w*Fra1 and *w*Fra2 of *Wolbachia* (Figs 1–3 and S2 Table). Mating experiments showed that crosses between *W*^+^ males and *W*^−^ females produced no offspring, indicating that CI occurred in *A*. *fraudatrix* (Fig 5). Screening experiments conducted using *Rickettsia*, *Spiroplasma*, *Arsenophonus and Cardinium* showed that neither the Fukaura nor the Shiwa population was not infected with these endosymbionts. Therefore, the CI observed in *A*. *fraudatrix* appeared to be due to *Wolbachia*. The present study is the first to show that *Wolbachia* alters reproduction in the longicorn beetle.

There have been only two reports of *Wolbachia* detection in the cerambycid beetles *M*. *alternatus* [28, 29] and *M*. *sartor* [24], and only the latter identified *Wolbachia* as a microbial presence. We believe that there are many difficult objectives with using longicorn beetles in *Wolbachia* research. First, it is difficult to collect a large number of individuals. In particular, forest longicorn beetles can only be found around a few weakened trees, which occur accidentally in forests, so it is difficult to obtain many individuals of various species. The second reason is the length of the generation time of cerambycid beetles. To clarify the effect of *Wolbachia* on host insects, it is necessary to rear the insects for several generations. However, many species of cerambycid beetle are univoltine, semivoltine, or multivoltine. *A*. *fraudatrix* populations that inhabit the northern part of Honshu Island in Japan (S1 Fig) have been shown to be semivoltine [31]. The long generation time of cerambycid beetles may not be suitable for use in *Wolbachia* studies. The third problem is the difficulty of rearing the insects. The lack of an established rearing method for the majority of cerambycid insects seriously impedes investigations of the effects of *Wolbachia* on the hosts. Rearing *A*. *fraudatrix* for successive generations in the laboratory is also extremely difficult. If the artificial diets for rearing *M*. *alternatus* could not have been applied to *A*. *fraudatrix* in the study, we would not have been able to clarify that *Wolbachia* caused CI in the insect. In order to extend *Wolbachia* research to longicorn beetles, it is necessary to develop methods that allow simple rearing of the insects indoors.

The *W*^+^ and *W*^−^ individuals of *A*. *fraudatrix* examined in this study were obtained from two localities in northern part of the Tohoku district in Japan. Fukaura is located on the coast of the Sea of Japan in Aomori Prefecture, whereas Shiwa is inland in Iwate Pref. (S1 Fig). The distance between the two towns is only 100 km. Therefore, if the Fukaura population of *A*. *fraudatrix* can move to the east, population replacement is expected to occur there, due to the strong CI caused by *Wolbachia* infecting the Fukaura population. In this district, however, the Ohu Mountain Range, composed of mountains with altitudes of 1,000–2,000 m above sea level, runs from north to south, dividing the area into west and east sides (S1 Fig). This geographical obstacle may function as barriers between the *A*. *fraudatrix* populations on the west and east sides, leading to differences in *Wolbachia* infection status. In the same area, previous molecular biology research concluded that *M*. *alternatus* spread beyond the Ohu Mountain Range has rarely occurred [53]. To demonstrate this inference in *A*. *fraudatrix*, a broader survey of natural insect populations covering the west and east sides of the mountain range is needed.

The evolutionary lineages of *Wolbachia* have been classified into at least 17 possible supergroups A–S, without G and R [52, 54]. Previous studies have shown that three populations of *M*. *sartor* in Europe harbored four strains of *Wolbachia*, all of which belonged to supergroup A [24]. The *Wolbachia* genes transferred onto the genome of *M*. *alternatus* were also included in supergroup A [28]. In this study, two strains of *Wolbachia*, *w*Fra1 and *w*Fra2, were detected in the Fukaura population of *A*. *fraudatrix*, and both of these strains were in supergroup A (Fig 1). All *Wolbachia* strains and genes detected from longicorn beetles to date belong to supergroup A.

It is very interesting that no singly-infected insect was found in the Fukaura population this time, and all were doubly-infected, although the sample size was relatively small. Although the reason why superinfection is stably inherited is unknown at this time, the combination of the two *Wolbachia* strains may cause strong CI expression, or each strain may play a different role that contributes to stable infection. In natural populations of the adzuki bean beetle *C*. *chinensis*, two *Wolbachia* strains, *w*BruCon and *w*BruOri, exhibit very high frequency of infection [55, 56]. The former causes complete CI, while the latter causes moderate CI in the host insect [56]. In somatic tissue of the host insect, the titers of *w*BruCon were higher than those of *w*BruOri, but in the nurse tissue of female, the titers of the latter were considerably higher than those of the former [57]. Based on the relationships between the intensities of CI and the bacterial titers in the nurse tissues of the two *Wolbachia* strains, Ijichi et al. [57] inferred the strategy of each strain for survival, reproduction, and transmission, as follows. The maintenance of *w*BruCon may be principally realized by the use of an efficient mechanism for CI. In contrast, the maintenance of *w*BruOri may be mainly attributed to a sophisticated mechanism for vertical transmission. Our study revealed that *A*. *fraudatrix* was infected with two strains of *Wolbachia*, and the titer of *w*Fra1 was about three-fold higher than that of *w*Fra2 in ovaries, although no difference in the bacterial titer between both strains was observed in muscle tissues (Fig 4). As in *C*. *chinensis*, the major difference in infection level in germ tissues in females of *A*. *fraudatrix* between the two strains may reflect the differences in their survival and transmission strategies. However, we could not determine the intensity of CI caused by *w*Fra1 or *w*Fra2 alone in the host insect in this study. The two strains of *Wolbachia* in *A*. *fraudatrix* could be removed by administering antibiotics to the host insect (Table 1). Hence, if an insect line with a single infection with either *w*Fra1 or *w*Fra2 could be produced by treatment with antibiotics, it would be possible to evaluate the intensity of CI caused by each *Wolbachia* strain.

In recent years, *Wolbachia* has attracted attention as an agent for the biological control of pest insects and vector-borne diseases. The idea is to artificially introduce *Wolbachia* from naturally carrying the bacterium into target insects that do not carry *Wolbachia*, so that the reproductive manipulations induced by *Wolbachia* are expressed in the new host insects. In this case, it is essential that the phenotype of reproductive manipulation is CI, leading to the suppression of populations by mass release of infected males [58–65] or replacement of populations with unique characteristics, such as pathogen blocking or fitness deficits, by the release of infected males and females [66–70]. In this study, the presence of *Wolbachia* causing CI in longicorn beetles was demonstrated. The most serious forest disease in the world involving longicorn beetles is thought to be pine wilt disease [27]. It has been reported that field populations of *M*. *alternatus* have not been infected with *Wolbachia* [28, 29]. Therefore, if the *Wolbachia* in *A*. *fraudatrix* can be introduced into *M*. *alternatus* of the same family, it may be possible to reduce the ability of *M*. *alternatus* to act as a vector insect, thorough population suppression or population replacement induced by *Wolbachia*. We hope that our findings will lead to new technological developments for the control of forest disease in the future.

## Supporting information

S1 Fig Locations of two towns (Fukaura and Shiwa) collected *A*. *fraudatrix* used in this study. (PDF)

S2 Fig Nucleotide sequences of two types of *wsp* gene derived from single individuals of the Fukaura population of *A*. *fraudatrix*, and each primer site used to detect them separately. (PDF)

S3 Fig Diagnostic PCR detection of the *ftsZ*, *wsp* and 16S rRNA genes of *Wolbachia* in two *A*. *fraudatrix* populations from Fukaura and Shiwa towns. M: Molecular size marker (100 bp DNA ladder).
(PDF)

S4 Fig PCR gel images of all individuals of *A*. *fraudatrix* used in antibiotic treatment. 1 and 2 show the *Wolbachia* strains *w*Fra1 and *w*Fra2, respectively. NC: a female from the Shiwa population (negative control); PC: a female from the Fukaura population (positive control); M: molecular size marker (100 bp DNA ladder).
(PDF)

S1 Table Primers for diagnostic PCR detection of *Rickettsia*, *Spiroplasma*, *Arsenophonus*, and *Cardinium*, and for *Wolbachia* MLST. (DOC)

S2 Table Results of diagnostic PCR detection of *Wolbachia*, *Rickettsia*, *Spiroplasma*, *Arsenophonus*, and *Cardinium* of 36 individuals from the Fukaura population of *A*. *furaudatrix*, and 10 from the Shiwa population. (XLSX)
