# Supplementary figures and images for: Cytoplasmic incompatibility in the semivoltine longicorn beetle Acalolepta fraudatrix (Coleoptera: Cerambycidae) double infected with Wolbachia
Source: PLoS One. 2022 Jan 14;17(1):e0261928. doi: 10.1371/journal.pone.0261928 (PMC8759696; doi:10.1371/journal.pone.0261928)

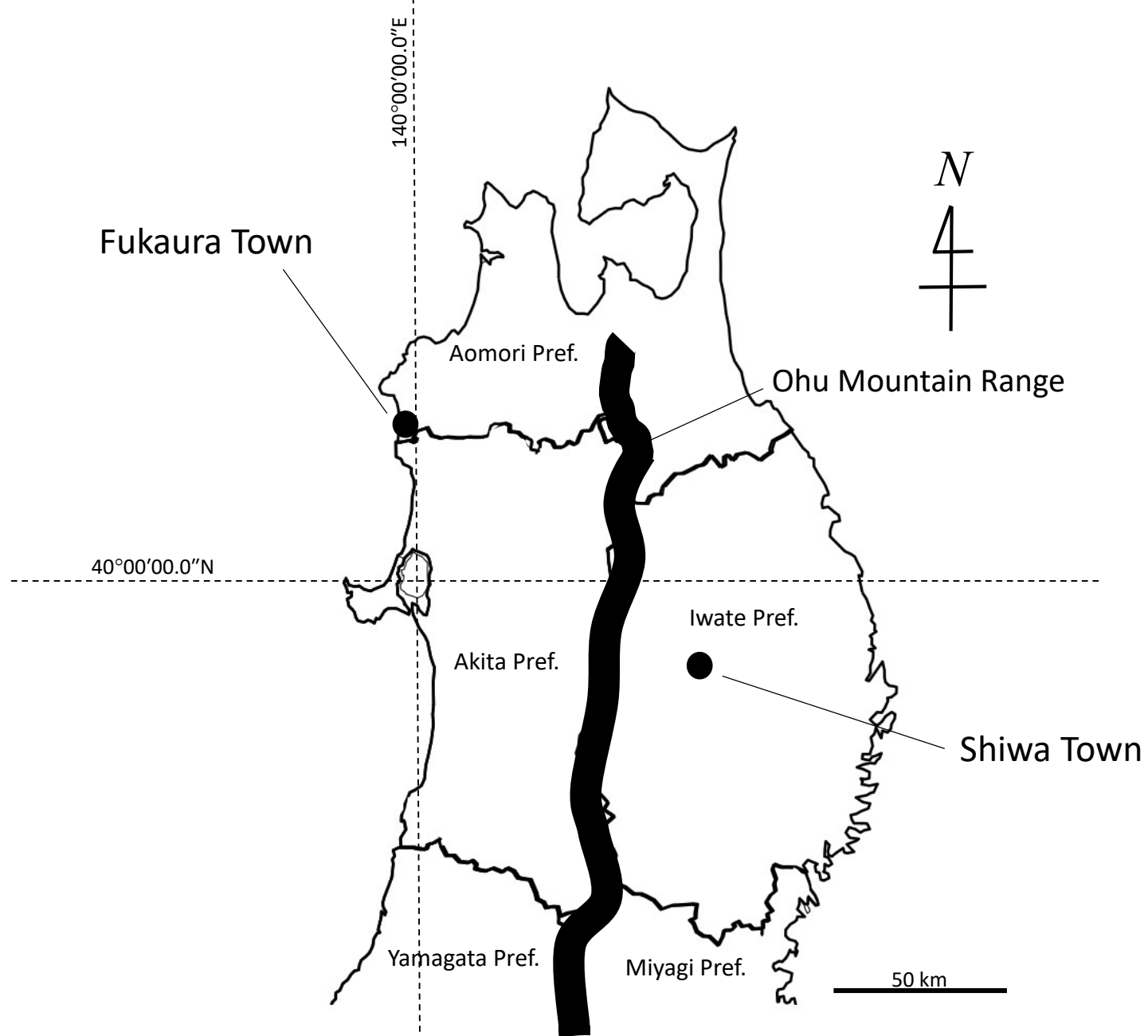

Northern part of Honshu island in Japan

Supplement: S1 Fig — (PDF) [file pone.0261928.s001.pdf]

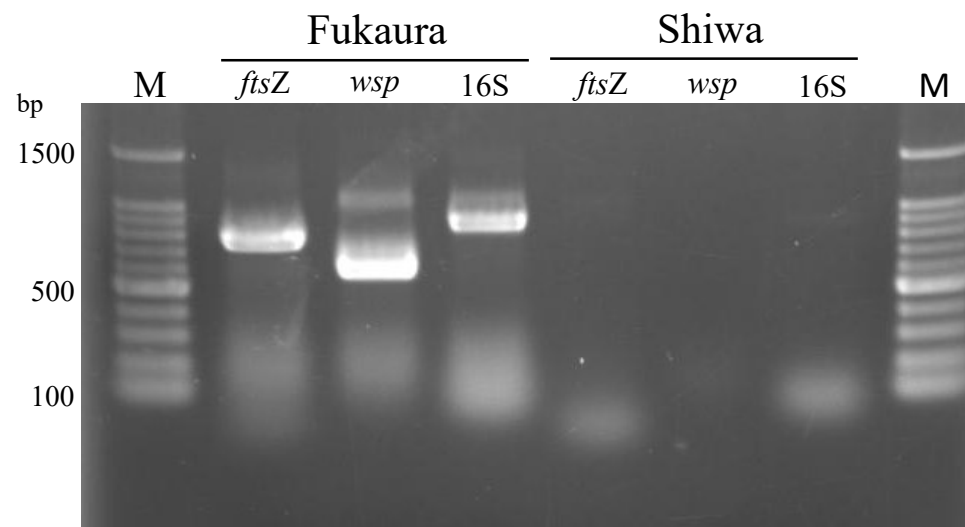

Supplement: S3 Fig — M: Molecular size marker (100 bp DNA ladder). (PDF) [file pone.0261928.s003.pdf]

Tetracycline  
concentration

0.5%

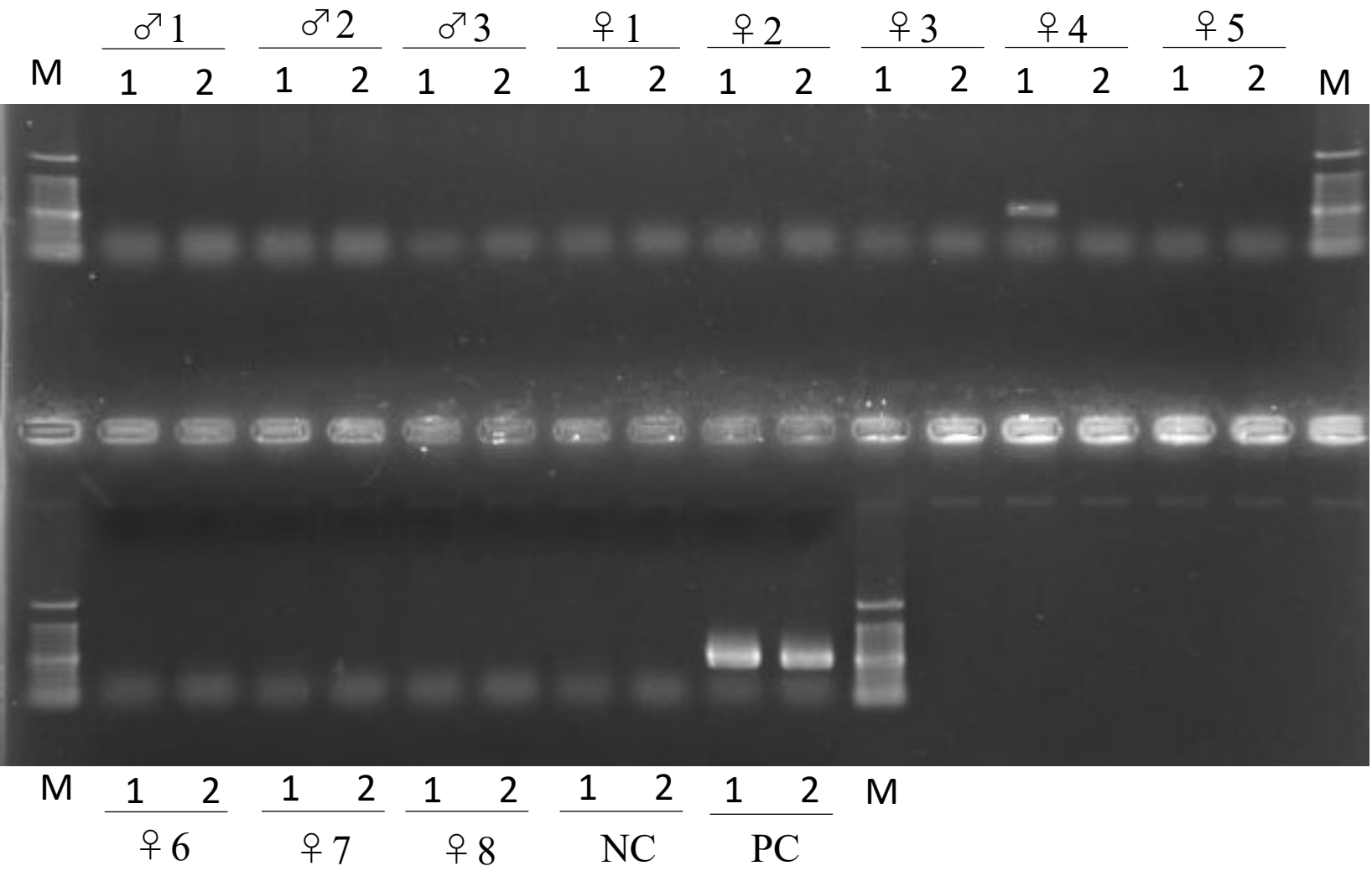

1.0%

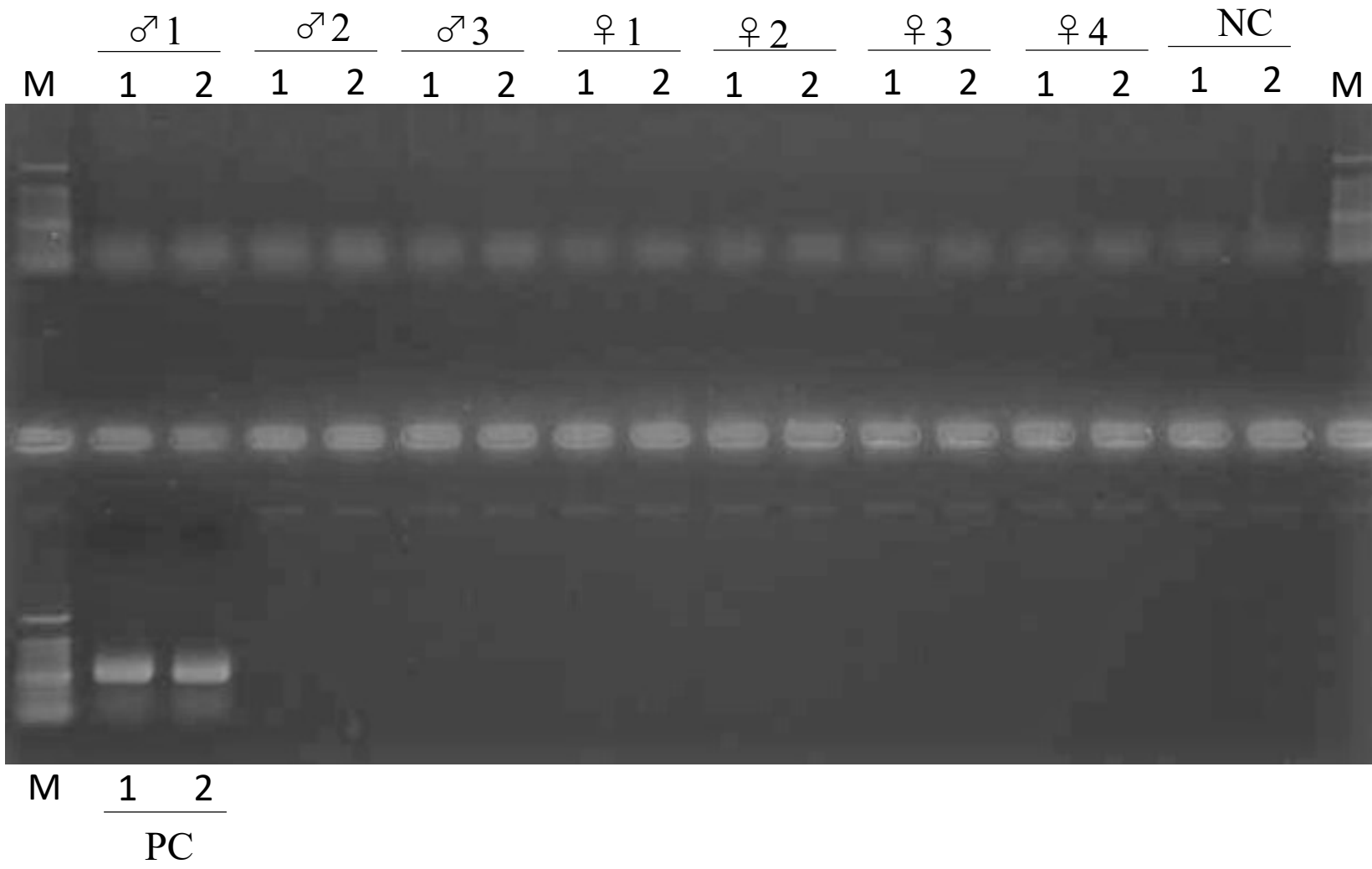

0%

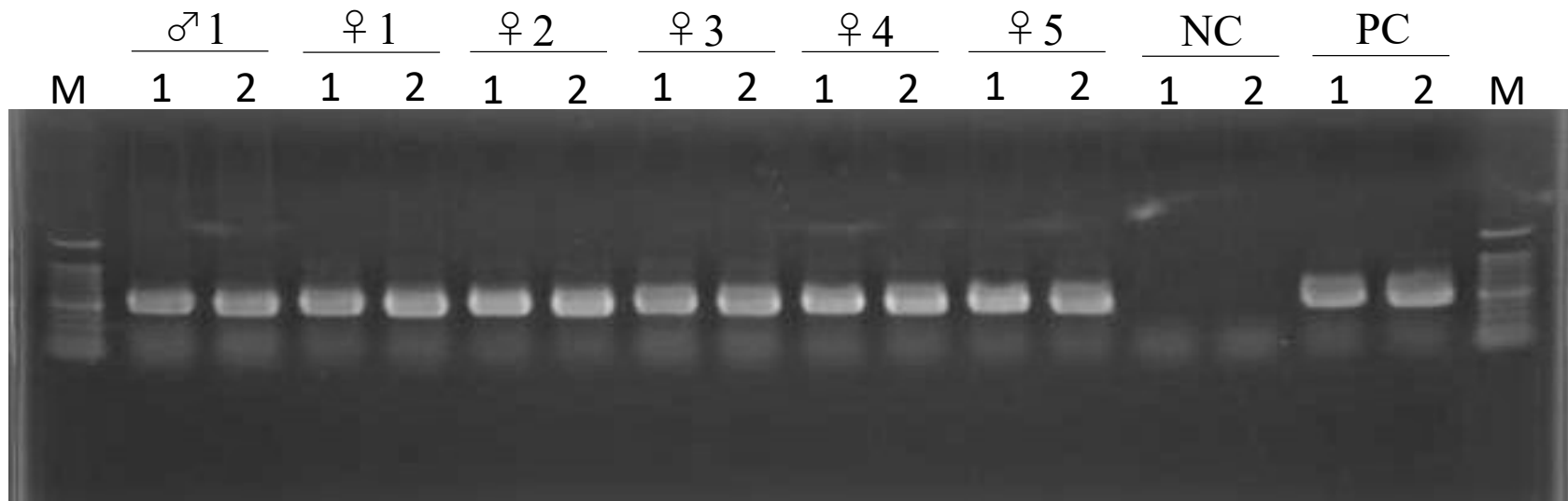

Supplement: S4 Fig — 1 and 2 show the Wolbachia strains wFra1 and wFra2, respectively. NC: a female from the Shiwa population (negative control); PC: a female from the Fukaura population (positive control); M: molecular size marker (100 bp DNA ladder). (PDF) [file pone.0261928.s004.pdf]
