# Supplementary material for: Cytoplasmic incompatibility in the semivoltine longicorn beetle Acalolepta fraudatrix (Coleoptera: Cerambycidae) double infected with Wolbachia
Source: PLoS One. 2022 Jan 14;17(1):e0261928. doi: 10.1371/journal.pone.0261928 (PMC8759696; doi:10.1371/journal.pone.0261928)
Supplement: S2 Fig — (PDF) [file pone.0261928.s002.pdf]

```
wFra1 81F→ TAGCTACTAC GTTCGTTTGC AATACAACGG TGAAATTTTA CCTCTTTTCA CAAAAGTTGA
wFra2      TAGCTACTAC GTTCGTTTGC AATACAACGG TGAAATTTTA CCTCTTTTCA CAAAAGTTGA

               wspwFra1_F
           ──────────────────→
wFra1      TGGTATTACC TATAAGAAAG ACAATAATGA TTACAGTCCA TTAAAAGCGT CTTTTATAGC
wFra2      TGGTATTGCA CATAAATCAG GCAAAGACAA TAATAGTCCC TTAAAAGCAT CTTTTATAGC
           ──────────────────→
               wspwFra2_F

wFra1      TGGTGGTGGT GCATTTGGTT ACAAAATGGA CGACATCAGG GTTGATGTTG AAGGGGTTTA
wFra2      TGGCGGTGGT GCGTTTGGTT ATAAAATGGA CGACATCAGG GTTGACGTTG AAGGACTTTA

                                             QwFra1_R
                                  ←──────────────────────
wFra1      TTCGCAACTA AGCAAAAATA ATGTTACAGG TGCAGCATTT AACCCAGATA CTGTTGCAGA
wFra2      CTCATGGTTG AATAAAGATG------CAGA TGTAGTAGGT ------GATA CAGTTGCAGA
                                ←────────────────────────────────────
                                             QwFra2_R

wFra1      CAGTTTAACA GCAATTTCAG GACTAGTTAA CGTTTATTAC GATATAGCAA TTGAAGATAT
wFra2      AAGCTTAACA GCAATTTCAG GATTAGTTAA CGTTTATTAC GATGTAGCGA TTGAAGACAT

wFra1      GCCTATCACT CCATATGTTG GTGTTGGTGT TGGTGCAGCG TATATTAGCA CACCTTTGGC
wFra2      GCCTATCACT CCATATGTTG GTGTTGGTGT TGGTGCAGCG TATATTAGCA CACCTTTGGC

wFra1      AACTGCTGTG AGTAGTCAAA ATGGTAAATT TGCTTTTGCT GGTCAAGCAA GAGCTGGTGT
wFra2      AACTGCTGTG AGTAGTCAAA ATGGTAAATT TGCTTTTGCT GGTCAAGCAA GAGCTGGTGT

wFra1      TAGTTACGAT GTAACTCCAG AAGTCAAACT TTACGCTGGA GCTCGCTATT TCGGTTCTTA
wFra2      TAGTTACGAT GTAACTCCAG GAGTCAAACT TTACGCTGGA GCTCGCTATT TCGGTTCTTA

wFra1      TGGTGCTAAC TTTGATAAAA CTGACAAAGA CGGCAAAGGG GAACTCAAAG TTCTTTACAG
wFra2      TGGTGCTAAC TTTGATAAAA CTGACAAAGA CGGCAAAGGG GAACTCAAAG TTCTTTACAG

wFra1      CACTGTTGGT GCAGAAGC← 691R
wFra2      CACTGTTGGT GCAGAAGC
```
