# Supplementary material for: Cytoplasmic incompatibility in the semivoltine longicorn beetle Acalolepta fraudatrix (Coleoptera: Cerambycidae) double infected with Wolbachia
Source: PLoS One. 2022 Jan 14;17(1):e0261928. doi: 10.1371/journal.pone.0261928 (PMC8759696; doi:10.1371/journal.pone.0261928)
Supplement: S1 Table — (DOC) [file pone.0261928.s005.doc]

**S1 Table. Primers for diagnostic PCR detection of *Rickettsia, Spiroplasma, Arsenophonus*, and *Cardinium*, and for *Wolbachia* MLST.**

| Target symbiont | Target gene | Primer name | Primer sequence (5’-3’) | Product size (kb) | Temp.^*^ (°C) | Refs. |
| --- | --- | --- | --- | --- | --- | --- |
| *Rickettsia* | 16S rRNA | Rick 112F | CTTGCTCCAATTAGTTAGTGGC | 0.1 | 62 | 41 |
|  |  | Rick16SR | CATCCATCAGCGATAAATCTTTC |  |  | 41 |
| *Spiroplasma* | 16S rRNA | TKSSspF | AAGCCTGATGGAGCAATGC | 0.1 | 62 | 41 |
|  |  | TKSSspR | TAGCCGTGGCTTTCTGGTAA |  |  | 41 |
| *Arsenophonus* | 16S rRNA | 16S A1 | AGAGTTTGATCMTGGCTCAG | 1 | 58 | 41 |
|  |  | Ars16SR | TTAGCTCCGGAGGCCACAGT |  |  | 41 |
| *Cardinium* | 16S rRNA | Car-sp-F | CGGCTTATTAAGTCAGTTGTGAAATCCTAG | 0.5 | 57 | 41 |
|  |  | Car-sp-R | TCCTTCCTCCCGCTTACACG |  |  | 41 |
| *Wolbachia* MLST | *gatB* | gatB_F1 | GAKTTAAAYCGYGCAGGBGTT | 0.45 | 54 | 44 |
|  |  | gatB_R1 | TGGYAAYTCRGGYAAAGATGA |  |  | 44 |
|  | *coxA* | coxA_F1 | TTGGRGCRATYAACTTTATAG | 0.48 | 54 | 44 |
|  |  | coxA_R1 | CTAAAGACTTTKACRCCAGT |  |  | 44 |
|  | *hcpA* | hcpA_F1 | GAAATARCAGTTGCTGCAAA | 0.5 | 54 | 44 |
|  |  | hcpA_R1 | GAAAGTYRAGCAAGYTCTG |  |  | 44 |
|  | *ftsZ* | ftsZ_F1 | ATYATGGARCATATAAARGATAG | 0.52 | 54 | 44 |
|  |  | ftsZ_R1 | TCRAGYAATGGATTRGATAT |  |  | 44 |
|  | *fbpA* | fbpA_F1 | GCTGCTCCRCTTGGYWTGAT | 0.5 | 59 | 44 |
|  |  | fbpA_R1 | CCRCCAGARAAAAYYACTATTC |  |  | 44 |

^*^Annealing temperature.
